# Time trends in HPV vaccination according to country background: a nationwide register-based study among girls in Norway

**DOI:** 10.1186/s12889-021-10877-8

**Published:** 2021-05-03

**Authors:** Randi Dalene Bjerke, Ida Laake, Berit Feiring, Geir Aamodt, Lill Trogstad

**Affiliations:** 1 Department of Public Health Science, Norwegian University of Life Sciences, Ås, Norway; 2 Division of Infection Control and Environmental Health, Norwegian Institute of Public Health, Oslo, Norway

**Keywords:** Human papillomavirus, Childhood immunisation programme, HPV vaccine, Immigrant background, Socioeconomic factors, Parental education, Income, Time trends

## Abstract

**Background:**

Since the human papillomavirus (HPV) vaccine was introduced in Norway in 2009, the vaccine uptake has increased. Whether this increase is similar regardless of the girls’ country background is unknown. We examined changes in HPV vaccine uptake from 2009 to 2014 and studied the impact of parental education and income on HPV vaccine uptake according to country background.

**Methods:**

Girls in the first six birth cohorts (1997–2002) eligible for HPV vaccination were identified through the National Registry. Information on HPV vaccination, country background and socioeconomic factors was extracted from the Norwegian Immunisation Registry and Statistics Norway. Risk differences (RDs) and confidence intervals (CIs) were estimated with linear binomial regression. A total of 177,387 girls were included in the study.

**Results:**

The HPV vaccine uptake increased from 72.5% in 2009 to 87.3% in 2014. The uptake increased for girls in all country background categories. Highest vaccine uptake was observed in girls with East−/South-East Asian background, 88.9% versus 82.5% in the total population. Vaccine uptake decreased slightly with increasing parental education, RD = − 1.6% (95% CI: − 2.3% to − 0.8%) for highest compared with lowest education level. In contrast, the uptake increased with increasing household income, RD = 4.9% (95% CI, 4.3 to 5.5%) for highest compared with lowest quintile. Parental education had largest impact in girls with Asian background, RD = − 8.1% (95% CI − 10.5% to − 5.6%) for higher vs lower education. The largest impact of household income was observed in girls with background from Middle East/Africa, RD for a 200,000 NOK increase in income was 2.1% (95% CI 1.2 to 3.0%).

**Conclusions:**

The HPV vaccine uptake differed with country background but increased over time in all country background categories. Moreover, the impact of education and income on vaccine uptake differed with country background.

**Supplementary Information:**

The online version contains supplementary material available at 10.1186/s12889-021-10877-8.

## Background

Infection with human papillomavirus (HPV) is the most common sexually transmitted infection. HPV-infection is a necessary cause of cervical cancer. In addition, there is evidence linking HPV-infection with cancers of the anus, vulva, vagina, penis, and oropharynx [1]. Since 2009, HPV vaccine has been offered free of charge to all Norwegian 12-years-old girls through a school-based programme within the Norwegian Childhood Immunisation Programme (NCIP) [2]. The quadrivalent vaccine, Gardasil®, was used from the introduction in autumn 2009. After a new tender, the bivalent vaccine Cervarix® has been used since autumn 2017. The HPV vaccination coverage is steadily increasing, but is still lower than for the other childhood vaccines offered through the NCIP. As of 2019, the vaccination coverage among 16-year-olds (born in 2003) is 89% for HPV vaccine (girls only), and 94% for the other vaccines [3].

In a previous study, we reported socioeconomic differences in HPV vaccine uptake among girls in Norway [4]. The proportion of girls initiating HPV vaccination increased with increasing maternal income. In contrast, high maternal education was associated with lower likelihood of initiating HPV vaccination. A Canadian study with data from a publicly funded school-based programme reported similar findings on education [5]. Moreover, differences in initiation of HPV vaccination according to country background have been reported in other countries with publicly funded immunisation programmes [6–9].

Since the HPV vaccine was introduced in the NCIP, the coverage has increased [3]. However, it is not known if the increase is similar regardless of the girls’ country background. Moreover, whether education and income can be of different importance depending on country background has not been assessed. The aim of the present study was to investigate changes over time in HPV vaccine uptake in Norway according to country background. Moreover, we examined whether the impact of parental education and income on HPV vaccine uptake differed with country background.

## Materials and methods

### Study design and data sources

The study is based on national registries. Information on dates of birth, immigration, emigration, and death was obtained from the National Registry. Information on HPV vaccination was obtained from the Norwegian Immunisation Registry. Information on maternal and paternal education level, household income in 2011, country of birth of the study participants and their parents, number of siblings, and county of residence was extracted from Statistics Norway. Data from the different registries were linked using the unique identification number assigned to all residents of Norway. The current study was approved by The Norwegian Regional Committee for Medical and Health Research Ethics, “Ref 2012/1619/REK Sør-Øst”.

### Study population

**Fig. 1:**
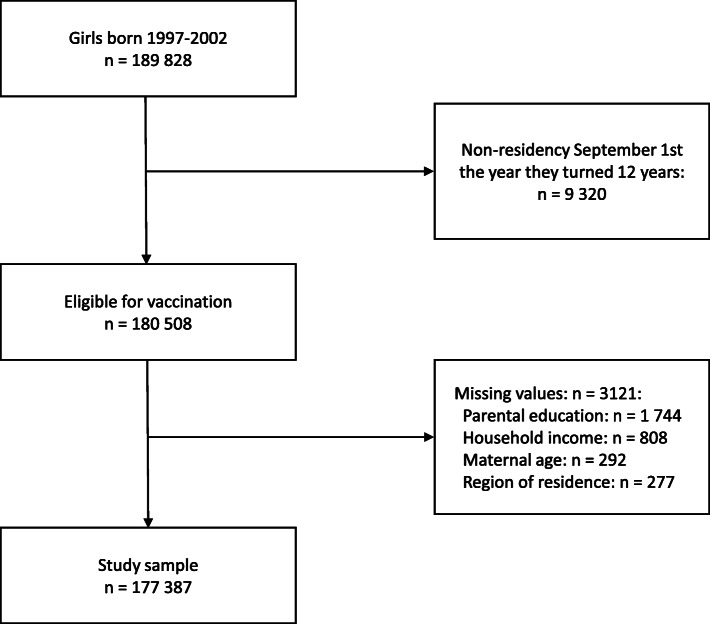
Flow chart of study population

Girls in the first six birth cohorts eligible for HPV vaccination through the NCIP (born 1997–2002) were identified through the National Registry, *n* = 189,828 (Fig. 1). Since we were only interested in girls who were offered the HPV vaccine, we excluded 9320 girls not residing in Norway on September 1, the year they turned 12, i.e. at the start of 7th grade when the vaccine was offered. Furthermore, we excluded girls with missing information on both maternal and paternal education, household income, maternal age, and region of residence, leaving 177,387 girls eligible for analysis.

### Outcome variable

All girls were offered three doses of HPV vaccine. The outcome variable ‘HPV vaccine uptake’ was defined as receipt of at least one dose of the HPV vaccine, as of June 19, 2015.

### Exposure variables

The main exposure was ‘Programme year’. Each programme year corresponds to an academic year running from 20 August to 20 June the following year [4]. During each programme year (2009–2014), the vaccine was offered to only one birth cohort. Thus, year of birth was used to allocate programme year. In the main analysis, programme year was treated as a categorical variable with the first year as the reference category.

‘Country background’ was defined as “Norwegian” if at least one parent was born in Norway. Otherwise, country background was defined as the girl’s country of birth if she was foreign born, or as her mother’s country of birth if she was born in Norway. Country background was categorised as: ‘Norway’, ‘Western Europe’, ‘Central- and Eastern Europe (including previous Soviet republics)’, ‘Middle East and North Africa’, ‘South-Asia’, ‘East−/South-East Asia’, ‘Sub-Saharan Africa’, and ‘America and Oceania’ (see Supplementary table) for countries in each category). In some analyses, the original eight categories were combined into the following four categories; ‘Norway’, ‘Europe, America, and Oceania’, ‘Middle East and Africa’, and ‘Asia’. The categorisation was based on the countries’ epidemiological similarity and geographic closeness.

‘Parental education’ was defined as maternal education level. If information on maternal education was missing, paternal education was used. Parental education was categorised into four categories: ‘Primary/ lower secondary school’ (≤ 10 years of schooling); ‘Upper secondary school’ (11–14 years of schooling); ‘Higher education, undergraduate level’ (14–17 years of schooling); and ‘Higher education, graduate level’ (≥ 18 years of schooling). We also used a two-category version of ‘Parental education’ with the categories lower education (≤ 14 years) and higher education (> 14 years).

‘Household income’, the household total gross income, was divided into quintiles. For all birth cohorts, we used household income from 2011. This was the most recent information on household income that was available in our data. Thus, for the youngest birth cohorts, we did not have information on household income from the year before vaccination. Moreover, the proportion of girls with missing household income was substantially lower for 2011 than for household income from the previous years, due to possible non-residency prior to 2011.

### Covariates

‘Number of siblings’ was categorised as 0, 1, 2, 3, and ≥ 4. ‘Maternal age at time of daughter’s birth’ was categorised into age categories ≤ 25, 26–30, 31–35, and > 35 years. ‘Region of residence’ was defined as Oslo, Eastern-Norway, Southern-Norway, Western-Norway, Mid-Norway, and Northern-Norway.

### Statistical analysis

We modelled the association between the main exposures (programme year/year of birth, country background, parental education, and household income) and the outcome variable (initiation of HPV vaccination), using linear binomial regression to estimate risk differences (RDs) and corresponding 95% confidence intervals (CIs). The multivariable model also included number of siblings, maternal age at daughter’s birth, and region of residence. These factors were considered potential confounders since they are associated with HPV vaccine uptake and with parental education and household income. The difference in earnings by educational level is smaller in Norway than in other high-income countries [10]. This is reflected in the weak correlation between parental education and household income observed in our data (Spearman’s rank correlation coefficient = 0.34). Thus, we were not concerned about collinearity between parental education and household income.

Changes in vaccine uptake over time by country background (with four categories) was examined by including interaction terms between programme year (continuous) and country background. Programme year was treated as a continuous variable in this analysis in order to limit the number of parameters in the model. The impact of parental education and household income by country background was assessed by including interaction terms between country background and parental education (higher compared to lower), and between country background and household income (continuous). In these analyses, we used a Poisson model with robust standard deviation to estimate RDs because the linear binomial model failed to converge [11]. In these models, RDs for each category of country background was calculated as the sum of the coefficient corresponding to the main effect and the coefficient corresponding to the appropriate interaction term.

All tests were two-sided, and *p* <  0.05 was considered statistically significant. Statistical analyses were performed using STATA/SE 15.0 (StataCorp. College Station, Texas USA).

## Results

### Characteristics of the study population

Of the 177,387 girls included in the study, the majority had Norway as country background (89.5%) (Table 1). The percentage of girls in each of the other country background categories was 1–2%. Upper secondary school (11–14 years) was the most common parental education level (37.6%), while 8.4% had parents with the highest education level (≥ 18 years of schooling). Median household income level was 901,243 NOK (IQR 656,053 NOK–1,268,988 NOK).

**Table 1:** Characteristics of the study population. Girls offered HPV vaccine during 2009–2014 (*n* = 177,387)

	n (%)
**HPV vaccine uptake**	
Initiated	146,403 (82.5)
Did not initiate	30,984 (17.5)
**Country background**^**a**^	
Norway	158,738 (89.5)
Western Europe	2072 (1.2)
Central- and Eastern Europe	3887 (2.2)
Middle East and North Africa	3729 (2.1)
South-Asia	3355 (1.9)
East−/South-East Asia	2236 (1.3)
Sub-Saharan Africa	2775 (1.6)
America and Oceania	595 (0.3)
**Year of birth (Programme year)**^**b**^	
1997 (2009)	30,209 (17.0)
1998 (2010)	29,719 (16.8)
1999 (2011)	30,100 (17.0)
2000 (2012)	30,098 (17.0)
2001 (2013)	28,932 (16.3)
2002 (2014)	28,329 (16.0)
**Parental education (years of schooling)**	
Primary school/compulsory level (≤ 10)	32,865 (18.5)
Upper secondary level (11–14)	66,711 (37.6)
Higher education, undergraduate level (14–17)	63,003 (35.5)
Higher education, graduate level (≥ 18)	14,808 (8.4)
**Household income quintile (NOK)**	
1 (≤ 575,319)	34,736 (19.6)
2 (575,320–811,300)	35,412 (20.0)
3 (811,301–988,227)	35,704 (20.1)
4 (988,228–1,251,798)	35,778 (20.2)
5 (≥ 1,251,799)	35,757 (20.2)
**Number of siblings**	
0	8203 (4.6)
1	66,237 (37.3)
2	62,468 (35.2)
3	24,561 (13.9)
≥ 4	15,918 (9.0)
**Maternal age at time of daughter’s birth (years)**	
≤ 25	38,939 (22.0)
26–30	64,254 (36.2)
31–35	51,947 (29.3)
> 35	22,247 (12.5)
**Region of residence**	
Oslo	15,889 (9.0)
Eastern-Norway	45,259 (25.5)
Southern-Norway	34,892 (20.1)
Western-Norway	39,283 (22.2)
Mid-Norway	24,948 (14.1)
Northern-Norway	17,116 (9.7)

*HPV* Human papillomavirus

^a^List of countries in each category is provided in the Supplementary table

^b^Each programme year, the vaccine was offered to only one birth cohort

### Uptake of HPV vaccine

A total of 146,403 (82.5%) girls initiated HPV vaccination (Table 1). Initiation of HPV vaccination increased from 72.5% among girls in the first programme year (born in 1997), to 87.3% among girls in the sixth programme year (born in 2002) (Table 2). In total, the HPV vaccine uptake was 82.6% among girls with Norwegian background and 81.6% among girls with non-Norwegian background. Overall, girls from East−/South-East Asia had the highest HPV vaccine uptake (88.9%).

**Table 2:** HPV vaccination uptake among girls offered HPV vaccine during 2009–2014 (*n* = 177,387)

	HPV vaccine uptake^**a**^ n (%)	Univariable model^**b**^		Multivariable model^**b,c**^	
		RD (95% CI)	***P***-value	RD (95% CI)	***P***-value
**Country background**^**d**^					
Norway	131,185 (82.6)	0 (Ref)		0 (Ref)	
Western Europe	1544 (74.5)	−8.1 (−10.1 to −6.2)	< 0.001	−7.9 (−9.7 to −6.1)	< 0.001
Central- and Eastern Europe	3098 (79.7)	−2.9 (−4.2 to − 1.7)	< 0.001	−3.3 (−4.6 to − 2.1)	< 0.001
Middle East and North Africa	3079 (82.6)	−0.1 (− 1.3 to 1.2)	0.91	0.8 (− 0.4 to 2.0)	0.20
South-Asia	2925 (87.2)	4.5 (3.4 to 5.7)	< 0.001	4.9 (3.8 to 5.9)	< 0.001
East−/South-East Asia	1987 (88.9)	6.2 (4.9 to 7.5)	< 0.001	5.4 (4.3 to 6.6)	< 0.001
Sub-Saharan Africa	2130 (76.8)	−5.9 (−7.4 to −4.3)	< 0.001	−3.4 (− 5.0 to − 1.8)	< 0.001
America and Oceania	455 (76.5)	−6.2 (−9.5 to − 2.7)	< 0.001	− 5.5 (− 8.8 to − 2.2)	0.001
**Year of birth (Programme year)**^**e**^					
1997 (2009)	21,896 (72.5)	0 (Ref)		0 (Ref)	
1998 (2010)	24,086 (81.1)	8.5 (7.9 to 9.2)	< 0.001	8.6 (8.0 to 9.3)	< 0.001
1999 (2011)	25,136 (83.5)	11.0 (10.4 to 11.7)	< 0.001	11.2 (10.5 to 11.8)	< 0.001
2000 (2012)	25,463 (84.6)	12.1 (11.5 to 12.8)	< 0.001	12.4 (11.7 to 13.0)	< 0.001
2001 (2013)	25,104 (86.8)	14.3 (13.7 to 14.9)	< 0.001	14.4 (13.8 to 15.1)	< 0.001
2002 (2014)	24,718 (87.3)	14.8 (14.1 to 15.4)	< 0.001	15.1 (14.5 to 15.7)	< 0.001
**Parental education (years of schooling)**					
Primary school/compulsory level	26,946 (82.0)	0 (Ref)		0 (Ref)	
Upper secondary level	55,157 (82.7)	0.7 (0.2 to 1.2)	0.007	−0.1 (−0.6 to 0.4)	0.64
Higher education, undergraduate level	52,132 (82.8)	0.8 (0.2 to 1.3)	0.004	−0.8 (−1.4 to − 0.3)	0.001
Higher education, graduate level	12,168 (82.2)	0.2 (−0.6 to 0.9)	0.63	−1.6 (−2.3 to − 0.8)	< 0.001
**Household income quintile (NOK)**					
1 (≤ 575,319)	27,687 (79.7)	0 (Ref)		0 (Ref)	
2 (575320–811,300)	28,874 (81.5)	1.8 (1.2 to 2.4)	< 0.001	1.4 (0.9 to 2.0)	< 0.001
3 (811301–988,227)	29,888 (83.7)	4.0 (3.4 to 4.5)	< 0.001	3.7 (3.1 to 4.2)	< 0.001
4 (988228–1,251,798)	29,972 (83.8)	4.1 (3.5 to 4.6)	< 0.001	4.2 (3.7 to 4.8)	< 0.001
5 (≥ 1,251,799)	29,982 (83.9)	4.1 (3.5 to 4.7)	< 0.001	4.9 (4.3 to 5.5)	< 0.001
**Number of siblings**					
0	6558 (80.0)	0 (Ref)		0 (Ref)	
1	55,421 (83.7)	3.7 (2.8 to 4.6)	< 0.001	2.9 (2.0 to 3.8)	< 0.001
2	52,142 (83.5)	3.5 (2.6 to 4.4)	< 0.001	2.5 (1.7 to 3.4)	< 0.001
3	19,825 (80.7)	0.8 (−0.2 to 1.8)	0.13	0.4 (− 0.5 to 1.4)	0.37
≥ 4	12,457 (78.3)	−1.7 (−2.8 to − 0.6)	0.002	−1.2 (− 2.3 to − 0.2)	0.02
**Maternal age at time of daughter’s birth (years)**					
≤ 25	32,515 (83.5)	0 (Ref)		0 (Ref)	
26–30	53,690 (83.6)	0.1 (−0.4 to 0.5)	0.81	−0.8 (−1.3 to − 0.4)	< 0.001
31–35	42,760 (82.3)	− 1.2 (− 1.7 to − 0.7)	< 0.001	−2.3 (− 2.8 to − 1.8)	< 0.001
> 35	17,438 (78.4)	−5.1 (−5.8 to −4.5)	< 0.001	−5.4 (−6.1 to −4.8)	< 0.001
**Region of residence**					
Oslo	12,968 (81.6)	0 (Ref)		0 (Ref)	
Eastern-Norway	37,721 (83.3)	1.7 (1.0 to 2.4)	< 0.001	1.1 (0.4 to 1.8)	0.001
Southern-Norway	28,671 (82.2)	0.6 (− 0.2 to 1.3)	0.13	0.1 (− 0.6 to 0.9)	0.69
Western-Norway	32,551 (82.9)	1.2 (0.5 to 2.0)	0.001	0.4 (−0.3 to 1.1)	0.28
Mid-Norway	20,321 (81.5)	−0.2 (− 0.9 to 0.6)	0.68	− 0.6 (− 1.3 to 0.2	0.15
Northern-Norway	14,171 (82.8)	1.2 (0.4 to 2.0)	0.005	0.7 (−0.1 to 1.5)	0.09

*HPV* human papillomavirus, *RD* risk difference, *CI* confidence interval

^a^ Receipt of at least one dose of HPV vaccine

^b^ Risk differences are estimated with linear binomial regression

^c^ The model included country background, year of birth, parental education level, household income, number of siblings, maternal age at time of

daughter’s birth, and region of residence

^d^ List of countries in each category is provided in the Supplementary table

^e^ Each programme year, the vaccine was offered to only one birth cohort

The uptake in girls with South-Asian, and East−/South-East Asian background was significantly higher than in girls with Norwegian background, multivariable RDs were 4.9% (95% CI: 3.8 to 5.9%) and 5.4% (95% CI: 4.3 to 6.6%), respectively (Table 2). Girls with background from Western-Europe, Central- and Eastern-Europe, Sub-Saharan Africa, and America and Oceania were significantly less likely to initiate HPV vaccination, as compared to girls with Norwegian background, multivariable RDs were − 7.9 (95% CI: − 9.7 to − 6.1), − 3.3 (95% CI: − 4.6 to − 2.1), − 3.4 (95% CI: − 5.0 to − 1.8), and − 5.5 (95% CI: − 8.8 to − 2.2), respectively.

Initiation of HPV vaccination decreased slightly with increasing parental education. Compared to girls with parents in the category primary school/compulsory, girls with parents in the categories ‘higher education, undergraduate level’ and ‘higher education, graduate level’ were significantly less likely to initiate HPV vaccination, with multivariable RDs − 0.8% (95% CI: − 1.4% to − 0.3%) and − 1.6% (95% CI: − 2.3% to − 0.8%), respectively (Table 2). In contrast, we found a positive association between household income and initiation of HPV vaccination. Compared to girls in household income quintile 1, girls in household income quintiles 2, 3, 4, and 5 were significantly more likely to initiate HPV vaccination, RDs were 1.4% (95% CI: 0.9 to 2.0%), 3.7% (95% CI: 3.1 to 4.2%), 4.2% (95% CI: 3.7 to 4.8%), and 4.9% (95% CI: 4.3 to 5.5%), respectively.

### Changes over time (2009–2014) in HPV vaccine uptake by country background

**Fig. 2:**
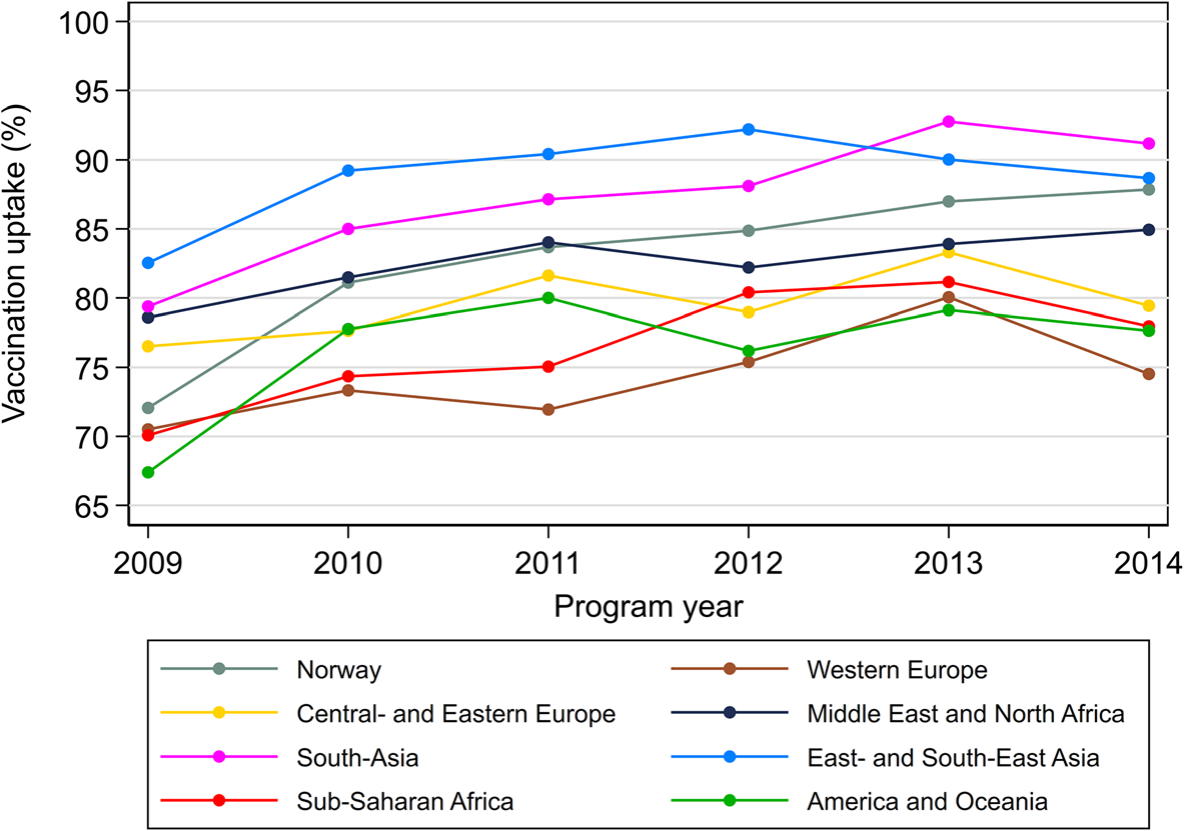
Uptake of at least one dose of HPV vaccine according to country of origin

Figure 2 shows the HPV vaccine uptake by programme year and country background. In all country background categories, the uptake increased from 2009 to 2014. Initially, girls with Norwegian background had a lower HPV vaccine uptake than girls with background from Central- and Eastern Europe, Middle East and North Africa, South-Asia, and East- and South-East Asia.

There was an overall significant interaction between programme year and country background (*p* <  0.001). The increase in HPV vaccine uptake was significantly higher for girls with Norwegian background than for girls with other country backgrounds (all *p* <  0.001), the multivariable RD corresponding to increase in HPV vaccine uptake per year was 2.7% (95% CI: 2.6 to 2.8%) for Norway, 1.0% (95% CI: 0.4 to 1.6%) for Europe/America/Oceania, 1.3% (95% CI: 0.7 to 1.8%) for Middle East/Africa, and 1.6% (95% CI: 1.2 to 2.1%) for Asia (Table 3).

**Table 3:** Increase in HPV vaccine uptake per year (2009–2014) according to country background

	HPV vaccine uptake^**a**^		Multivariable model^**b**^	
	2009 n (%)	2014 n (%)		
			RD (95% CI)	*P*-value
**Country background**^**c**^				
Norway	19,706 (72.1)	22,023 (87.9)	2.7 (2.6 to 2.8)	< 0.001
Europe, America, and Oceania	684 (73.7)	936 (77.8)	1.0 (0.4 to 1.6)	0.001
Middle East and Africa	755 (75.2)	954 (81.8)	1.3 (0.7 to 1.8)	< 0.001
Asia	751 (80.7)	805 (90.2)	1.6 (1.2 to 2.1)	< 0.001

*HPV* Human papillomavirus, *RD* Risk difference, *CI* Confidence interval

^a^Receipt of at least one dose of HPV vaccine

^b^Risk differences are estimated with linear binomial regression. The model included country background, year of birth (corresponding to programme year), parental education level, household income, number of siblings, maternal age at time of daughter’s birth, region of residence, and interaction terms between country background and year of birth. RDs correspond to the change in HPV vaccine uptake per year

^c^List of countries in each category is provided in the Supplementary table

### Impact of higher parental education by country background

Girls with Norwegian background were more likely to have parents with higher education (46.0%) than girls with background from Europe, America, and Oceania (39.3%), Middle East and Africa (17.2%), and Asia (19.5%) (Table 4). We found a significant interaction between parental education and country background (*p* <  0.001). Higher parental education was significantly associated with lower vaccine uptake among girls in all country background categories. However, the association was weak among girls with Norwegian background, RD was − 0.6% (95% CI: − 1.0% to − 0.2%). The association was stronger for girls with country background Asia or Europe/America/Oceania, RDs were − 8.1% (95% CI: − 10.5% to − 5.6%) and − 7.8% (95% CI: − 9.9% to − 5.6%), respectively. For girls with Middle Eastern and African background the RD was − 2.7% (95% CI: − 5.3 to − 0.02).

**Table 4:** Impact of parental education on HPV vaccine uptake according to country background

	Higher education n (%)	HPV vaccine uptake^**a**^		Multivariable model^**b**^	
		Lower education n (%)	Higher education n (%)		
				RD (95% CI)^**b**^	***P***-value
**Country background**^**c**^					
Norway	73,023 (46.0)	70,561 (82.3)	60,624 (83.0)	− 0.6 (−1.0 to − 0.2)	0.005
Europe, America, and Oceania	2576 (39.3)	3199 (80.4)	1898 (73.7)	−7.8 (− 9.9 to − 5.6)	< 0.001
Middle East and Africa	1121 (17.2)	4328 (80.4)	881 (78.6)	−2.7 (−5.3 to − 0.02)	0.048
Asia	1091 (19.5)	4015 (89.2)	897 (82.2)	−8.1 (−10.5 to −5.6)	< 0.001

*HPV* Human papillomavirus, *RD* Risk difference, *CI* Confidence interval

^a^ Receipt of at least one dose of HPV vaccine

^b^ Risk differences are estimated with linear binomial regression. The model included country background, year of birth, parental education level, household income, number of siblings, maternal age at time of daughter’s birth, region of residence, and interaction terms between country background and parental education level. RDs correspond to the difference in HPV vaccine uptake between girls with higher parental education and girls with lower parental education

^c^ List of countries in each category is provided in the Supplementary table

### Impact of higher household income, by country background

Median household income (in NOK) was 930,136 for girls with Norwegian background, 685,123 for girls with European, American, and Oceanian background, 534,160 for girls with Middle Eastern and African background, and 698,839 for girls with Asian background (Table 5). We found a significant interaction between household income and country background (*p* <  0.001). There was a significant association between household income and initiation of HPV vaccination among girls with Norway and Middle East/Africa as country background. However, the association was weak among girls with Norwegian background, the RD for a 200,000 NOK increase in income was 0.2% (95% CI: 0.1 to 0.4%). The association was stronger for girls with Middle East/Africa as country background, RD = 2.1% (95% CI: 1.2 to 3.0%). Household income was not significantly associated with initiation of HPV vaccination for girls with Asia or Europe/America/Oceania as country background.

**Table 5:** Impact of household income on HPV vaccine uptake according to country background

	Household income,	Multivariable model^**a**^	
	median (NOK)	RD (95% CI)	***P***-value
**Country background**^**b**^			
Norway	930,136	0.2 (0.1 to 0.4)	< 0.001
Europe, America, and Oceania	685,123	− 0.2 (− 0.6 to 0.3)	0.44
Middle East and Africa	534,160	2.1 (1.2 to 3.0)	< 0.001
Asia	698,839	−0.4 (− 1.0 to 0.2)	0.19

*HPV* Human papillomavirus, *RD* Risk difference, *CI* Confidence interval

^a^ Risk differences are estimated with linear binomial regression. The model included country background, year of birth, parental education level, household income, number of siblings, maternal age at time of daughter’s birth, region of residence, and interaction terms between country background and household income. RDs correspond to the difference in HPV vaccine uptake between households with a 200,000 NOK difference in household income

^b^ List of countries in each category is provided in the Supplementary table

## Discussion

In this nationwide registry-based study, we studied the impact of country background, parental education, and household income on initiation of HPV vaccination in 12-year-old Norwegian girls between 2009 and 2014. We found an increase in HPV vaccine uptake over time among all girls regardless of country background. While girls with Asian background had the highest total HPV vaccine uptake, girls with Norwegian background had the highest increase in HPV vaccine uptake per year. Higher parental education was negatively associated with HPV vaccine uptake in all country background categories, but the association was weak for girls with Norwegian background. In contrast, higher household income was positively associated with HPV vaccine uptake. However, this was only significant among girls with Norwegian and Middle East/African backgrounds.

We found an increase in initiation of HPV vaccination over time among all categories of country background. Girls with Norwegian background started with a lower HPV vaccine uptake than several of the other country background categories. However, girls with Norwegian background had the highest increase per year. Possible explanations for this might be increased information and more positive attitudes towards the HPV vaccine. Increased information and positive focus on the vaccine may have had a more positive effect on Norwegian parents leading to greater willingness to accept vaccination compared to immigrant parents. Preceding the introduction of the HPV vaccine, Norwegian parents may have been more influenced by the heavy, negative discussions in the media and therefore more reluctant to vaccinate their daughters during the first programme years [12–14]. This would be in line with recent findings in a Danish study [15] that found that the decline in uptake following the public debate on the safety of the HPV vaccine in Denmark was less pronounced in immigrants and descendants of immigrants compared to native Danes, and suggested that these groups may be less influenced by the negative public debate than native Danes. Another Danish study examined the relation between a lower HPV vaccine uptake and increased media coverage in Denmark, mainly regarding suspected adverse events, such as POTS, following HPV vaccination [16]. Findings from the study indicate that this may have contributed to a lower HPV vaccine uptake among girls eligible for vaccination between 2013 and 2016 in Denmark. However, these claims do not appear to have affected the HPV uptake in Norway to a large extent.

Previous studies have reported a lower HPV vaccine uptake among girls with immigrant background [6, 7, 9, 15, 17]. A Scottish cross-sectional study found the HPV vaccine uptake to be significantly lower for girls with Polish background, as compared to girls from the United Kingdom [18]. Studies conducted in Denmark and Sweden, with publicly funded HPV vaccine programmes, reported a lower proportion of girls initiating HPV vaccination among girls with immigrant background [6–9].

In the present study, girls with country background from Western Europe and Central−/Eastern Europe had a lower likelihood of initiating HPV vaccination, as compared to girls with Norwegian background. This is in line with previous findings from Denmark [8]. The lower uptake among these girls reflects the uptake in their countries of origin [19].

We observed that girls from Sub-Saharan Africa, America, and Oceania, were less likely to initiate HPV vaccination, as compared to Norwegian girls. Lower initiation of HPV vaccination among ethnic minorities has been related to integration and language barriers as well as cultural norms and religious beliefs [17, 20]. Information about the HPV vaccine in several languages (Arabic, English, French, Northern Sami, Polish, Russian, Somali, Spanish, Tigrinya, and Urdu) is available on the Norwegian Institute of Health’s website and for school health nurses to use when informing parents prior to vaccination [2, 21]. A Canadian systematic review found that cultural norms, knowledge gaps, and anti-vaccination beliefs were barriers to vaccinations [22]. These factors might be possible explanations for the lower HPV vaccine uptake among girls from Sub-Saharan Africa, America, and Oceania. However, our data do not include information on parental attitudes or beliefs.

The highest probability of initiating HPV vaccination was found among girls with country backgrounds from Asia. To our knowledge, a higher uptake among Asian girls, compared to non-immigrants, has not been observed in other countries than Norway and Denmark [15, 23]. The nationwide Danish study by Hertzum et al. [15], found that daughters of immigrants from Mid- and Eastern Asia had a higher HPV vaccine uptake as compared to daughters of native Danes (87% vs. 85%). Possible explanations for the findings in our study might be that Asian parents residing in Norway generally have positive attitudes towards the HPV vaccine, and that they consider it a privilege that the vaccine is offered free of charge. In our study, the majority of girls in the category East−/South-East Asia had Vietnam as country background (42%). In Vietnam, the HPV vaccine has not yet been included in the immunisation programme [24]. However, the vaccination coverage for other vaccines offered through the Vietnamese immunisation programme is high, and has increased from 2000 to 2015 [25, 26]. Hence, a high vaccination coverage in Vietnam might have positively affected attitudes among Vietnamese parents’ residing in Norway.

Despite a weak association, we found that the likelihood of initiating HPV vaccination decreased with increasing parental education level, which is similar to a Canadian study with data from a publicly funded school-based programme [5]. These findings are somewhat surprising, because it has been reported that highly educated people are more receptive to health information and use of health services, and make more active health related choices, as compared to people with lower education [27]. Both a Swedish and a Danish study reported an increased likelihood of initiation of HPV vaccination with increasing maternal education level [6, 7]. In Norway, parents with higher education may be more cautious towards the HPV vaccine than parents with only compulsory schooling, but this could not be assessed in our study since we did not have information on parental attitudes.

The negative association between higher parental education level and initiation of HPV vaccination was observed among girls in all categories of country background. Moreover, the association was stronger among girls with non-Norwegian background, as compared to girls with Norwegian background. To our knowledge, this is the first study to evaluate potential differences according to country background of the impact of parental education on HPV vaccine uptake. The stronger association among girls with non-Norwegian background might be explained by larger variations in educational attainment among parents with different country backgrounds. The proportion with higher parental education was 46% for girls with Norwegian background, but only 13% for girls with background from Sub-Saharan Africa. A reasonable explanation might be that a larger proportion of higher parental education leads to smaller differences in initiation of HPV vaccination.

In contrast to the negative association between higher parental education level and initiation of HPV vaccination, the proportion of girls initiating HPV vaccination increased with increasing household income. This is in line with two previous studies from low- and middle-income countries that reported that parental income was a barrier to achieving a sufficient childhood vaccination coverage [28, 29], as well as a Danish study, that reported that high maternal income was associated with higher probability of initiating HPV vaccination [6].

We also found significant interactions between household income and country background. The association was strongest for Middle East/Africa. A possible explanation might be related to differences in income between different categories of country background. Median household income was lowest for Middle East/Africa. A 200,000 NOK increase in income is a larger relative increase in categories with low income, and therefore the effect might be larger among these categories. To our knowledge, this is the first study to report that the impact of parental income in initiation of HPV vaccination differs with country background.

A major strength of this study is the large sample size and the diversity of the study participants. Our study includes individual data from different population-based registries, covering the total population. Hence, we were able to include girls from six birth cohorts eligible for HPV vaccination, as well as controlling for numerous confounders. A high proportion of girls were included in the study population (93.4%), thereby limiting selection bias. Another strength of using national registries, is that our study does not rely on self-reported data, which contributes to high quality and less misclassifications of outcomes and exposures. Notification to the Norwegian Immunisation Registry is mandatory for all vaccinations provided within the childhood immunisation programme. Moreover, vaccinations received abroad are also notifiable to the immunisation registry [30]. Thus, the potential misclassification due to vaccination abroad before arrival in Norway is limited.

Still, the present study also has limitations. With such a large dataset, we might detect significant results for clinically insignificant differences. Thus, results should be interpreted with caution. In order to detect a difference of 3 percentage points between the lowest income quintile and any other quintile with 90% power, we would need only 3519 individuals in each income quintile (10% of the number of girls in our study), if we assume that the vaccine uptake is 80% in the lowest income quintile and that the association between income and vaccine uptake is positive. Moreover, we would need 112,193 girls with Norwegian background and 1571 girls with East−/South-East Asian background (71% of the number of girls in our study) to detect a difference in vaccine uptake of 3 percentage points between these two groups with 90% power, if we assume that the vaccine uptake is 83% among girls with Norwegian background, that the ratio between the number of girls with Norwegian background and East/South-East Asian background is the same as in our study, and that the vaccine uptake is higher among girls with East−/South-East Asian background. For the last programme year (2014) there may be some delay in registration of vaccination in the immunisation registry [31] which may explain an apparent decline in the HPV uptake in 2014. Moreover, a larger proportion of girls with missing information on parents’ education, as well as income, did not have Norway as country background. Also, the study only has income data from 2011, and we were not able to assess the significance of change in income over time. Despite a small difference, the 3121 (1.7%) girls excluded due to missing information, were slightly less likely to initiate HPV vaccination, than girls in the final study population (82.3% vs 82.5%). This could be a limitation, due to the small proportion of girls in other country categories than Norway.

## Conclusions

In summary, the present study found inequalities in the uptake of the HPV vaccine related to both country background and socioeconomic factors in the publicly funded school-based programme in Norway.

Our findings are encouraging; girls in all country background categories experienced a higher HPV vaccine uptake in 2014 than in 2009. Nevertheless, the vaccine uptake still differs with country background. Our observations indicate that education and income are important predictors of disparities in HPV vaccine uptake. Moreover, these factors differ with country backgrounds.

## Supplementary Information

**Additional file 1: Supplementary table.** Countries in each category of country background.

## Data Availability

The data analyzed during this study consist of sensitive information on an individual level. Due to protection of privacy and restrictions from the Norwegian Data Inspectorate and the Regional Committee for Medical and Health Research Ethics, the data cannot be made publicly available.

## Abbreviations

CI: Confidence interval
HPV: Human papillomavirus
NCIP: Norwegian Childhood Immunisation Programme RD Risk difference
