# Supplementary material for: Time trends in HPV vaccination according to country background: a nationwide register-based study among girls in Norway
Source: BMC Public Health. 2021 May 3;21:854. doi: 10.1186/s12889-021-10877-8 (PMC8091748; doi:10.1186/s12889-021-10877-8)
Supplement: Supplementary file 1 — Additional file 1: Supplementary table. Countries in each category of country background. [file 12889_2021_10877_MOESM1_ESM.pdf]

Supplementary table. Countries in each category of country background

Category	Country
**Western Europe**	Andorra, Austria, Belgium, Cyprus, Denmark, Faroe Islands, Finland, France, Gibraltar, Germany, Greece, Greenland, Iceland, Ireland, Italy, Liechtenstein, Luxembourg, Malta, Monaco, The Netherlands, Portugal, San Marino, Spain, Sweden, Switzerland, United Kingdom
**Eastern Europe (including previous Soviet republics)**	Albania, Armenia, Azerbaijan, Belarus, Bosnia-Herzegovina, Bulgaria, Czech Republic, Croatia, Estonia, Georgia, Hungary, Latvia, Lithuania, Kazakhstan, Kirgizstan, Kosovo, Macedonia, Moldova, Montenegro, Poland, Romania, Russia Serbia, Slovakia, Slovenia, Ukraine, Tajikistan, Turkmenistan, Uzbekistan
**Middle East and North Africa**	Algeria, Bahrain, Egypt, Iran, Iraq, Israel, Jordan, Kuwait, Lebanon, Libya, Morocco, Oman, Palestine, Qatar, Syria, Saudi Arabia, Tunisia, Turkey, United Arab Emirates, Western Sahara, Yemen
**South Asia**	Afghanistan, Bangladesh, Bhutan, India, Maldives, Nepal, Pakistan, Sri Lanka
**East-/Southeast Asia**	Brunei Darussalam, Cambodia, China, Hong Kong, Indonesia, Japan, North Korea, South Korea, Laos, Macao, Malaysia, Mongolia, Myanmar, The Philippines, Singapore, Taiwan, Thailand, East Timor, Vietnam
**Sub-Saharan Africa**	Angola, Benin, Botswana, Burundi, Burkina Faso, Cameroon, Cape Verde, Central African Republic, Chad, The Comoros, Congo, Congo-Brazzaville, Djibouti, Eritrea, Ethiopia, Equatorial Guinea, Ivory Coast, Gabon, The Gambia, Ghana, Guinea, Guinea Bissau, Kenya, Zaire, Lesotho, Liberia, Madagascar, Mali, Malawi, Mauritania, Mauritius, Namibia, Niger, Nigeria, Mozambique, Mayotte, Reunion, Zimbabwe, Rwanda, Sao Tome and Principe, Senegal, Sierra Leone, Somalia, South Africa, South Sudan, Tanzania, Togo, Uganda, British Indian

	Ocean Territory, Seychelles, Sudan, Swaziland, St Helena, Zambia
**America and Oceania**	Australia, Canada, USA, New Zealand, US Virgin Islands, Barbados, Antigua and Barbuda, Belize, Bahamas, Bermuda, British Virgin Islands, Cayman Islands, Costa Rica, Cuba, Dominica, Dominican Republic, Grenada, Guadeloupe, Guatemala, Haiti, Honduras, Jamaica, Martinique, Mexico, Montserrat, Aruba, Nicaragua, Panama, El Salvador, Saint Kitts and Nevis, Saint Lucia, Saint Vincent and the Grenadines, Trinidad and Tobago, Puerto Rico, Argentina, Bolivia, Brazil, Guyana, Chile, Colombia, Ecuador, Falkland Islands, French Guyana, Paraguay, Peru, Surinam, Uruguay, Venezuela, American Samoa, Solomon, Islands, Christmas Island, Cook Islands, Fiji, Vanuatu, Tonga, French Polynesia, Kiribati, Tuvalu, Micronesia, Papua New Guinea, Samoa, New Caledonia, Palau
